# Locally common graphs

**DOI:** 10.1002/jgt.22881

**Published:** 2022-09-02

**Authors:** Endre Csóka, Tamás Hubai, László Lovász

**Affiliations:** ^1^ Combinatorics and Applications Research Division Alfréd Rényi Institute of Mathematics Budapest Hungary; ^2^ Department of Computer Science, Institute of Mathematics Eötvös Loránd University Budapest Hungary

**Keywords:** graph homomorphisms, graph theory

## Abstract

Goodman proved that the sum of the number of triangles in a graph on n nodes and its complement is at least $n^{3}∕24$; in other words, this sum is minimized, asymptotically, by a random graph with edge density 1/2. Erdős conjectured that a similar inequality will hold for $K_{4}$ in place of $K_{3}$, but this was disproved by Thomason. But an analogous statement does hold for some other graphs, which are called *common graphs*. Characterization of common graphs seems, however, out of reach. Franek and Rödl proved that $K_{4}$ is common in a weaker, local sense. Using the language of graph limits, we study two versions of locally common graphs. We sharpen a result of Jagger, Štovíček and Thomason by showing that no graph containing $K_{4}$ can be locally common, but prove that all such graphs are weakly locally common. We also show that not all connected graphs are weakly locally common.

## 1 INTRODUCTION

Let inj(F,G) denote the number of embeddings of the graph F in graph G. The following inequality was proved by Goodman [7]:

(1) $$\text{inj}\left(K_{3},G\right)+\text{inj}\left(K_{3},\bar{G}\right)\geq \frac{1}{4}|V\left(G\right){|}^{3},$$

where equality holds asymptotically if G is a random graph with edge density 1/2. (i.e., for the random graph $G_{n}=G\left(n,\frac{1}{2}\right)$ the ratio 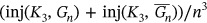 stochastically converges to $\frac{1}{4}$.) Erdős conjectured that a similar inequality will hold for $K_{4}$ in place of $K_{3}$, but this was disproved by Thomason [20] (see also Thomason [21] for a more “conceptual” proof). More generally, one can ask which graphs F satisfy

(2) $$\text{inj}\left(F,G\right)+\text{inj}\left(F,\bar{G}\right)\geq \left(1+o\left(1\right)\right)2^{1-|E\left(F\right)|}|V\left(G\right){|}^{|V\left(F\right)|}$$

for every graph G, where the o(1) refers to ∣V(G)∣→∞. Such graphs F are called *common graphs*. So the triangle is common, but $K_{4}$ is not. (Throughout the paper, we are going to assume that the graphs are simple and, unless stressed otherwise, have no isolated nodes.)

Many classes of bipartite graphs are known to be common, and the Sidorenko–Simonovits conjecture would imply that every bipartite graph is common. Among nonbipartite graphs, very few are known to be common. Franek and Rödl [6] proved that by deleting an edge from $K_{4}$ we get a common graph. More recently Hatami et al. [9] proved that the 5‐wheel is common, thus providing the first common graph with chromatic number 4. In the opposite direction, Jagger et al. [10] proved that no graph containing $K_{4}$ is common.

It will be more convenient to count homomorphisms instead of embeddings or copies of F. Let hom(F,G) denote the number of homomorphisms from F into G. We are interested in the case when ∣V(G)∣→∞, when $\text{inj}\left(F,G\right)=\text{hom}\left(F,G\right)+O\left(|V\left(G\right){|}^{|V\left(F\right)|-1}\right)$, and so we could replace inj by hom in the definition of common graphs (2). It will be even better to consider the normalized version $t\left(F,G\right)=\text{hom}\left(F,G\right)∕|V\left(G\right){|}^{|V\left(F\right)|}$, which can be interpreted as the probability that a random map ϕ:V(F)→V(G) preserves adjacency. With this notation, common graphs are those graphs F for which

$$t\left(F,G\right)+t\left(F,\bar{G}\right)\geq \left(1+o\left(1\right)\right)2^{1-|E\left(F\right)|}$$

for simple graphs G as ∣V(G)∣→∞.

Sidorenko [18] studied various “convexity” properties of graphs, one of which is closely related to common graphs. Let us say that a graph F has the *Sidorenko property* if for every graph G,

(3) $$t\left(F,G\right)\geq t{\left(K_{2},G\right)}^{|E\left(F\right)|}.$$

It is easy to see that nonbipartite graphs do not have this property, and Sidorenko conjectured that all bipartite graphs do. A closely related conjecture, in a different language, was formulated earlier by Simonovits [19]. For us, the significance of this study is that *the Sidorenko property implies that the graph is common* because of the following simple calculation.

(4) $$t\left(F,G\right)+\left(F,\bar{G}\right)\overset{\left(3\right)}{\geq }t{\left(K_{2},G\right)}^{|E\left(F\right)|}+{\left(1-t\left(K_{2},G\right)\right)}^{|E\left(F\right)|}\geq 2\cdot {\left(\frac{1}{2}\right)}^{|E\left(F\right)|}=2^{1-|E\left(F\right)|},$$

So the Sidorenko–Simonovits conjecture would imply that all bipartite graphs are common. Sidorenko's conjecture has been proved for several rather broad classes of bipartite graphs[4, 5, 11]; for a description of these classes, we refer to these publications and the references therein.

Franek and Rödl [6] proved that $K_{4}$ is common in a “local” sense: the original conjecture of Erdős said that the number of $K_{4}$'s in a graph and in its complement is minimized asymptotically by a random graph, and Franek and Rödl showed that this is true at least for graphs coming from a random graph by a small perturbation. A more natural formulation of this result was given in [13], using notions of graph limit theory (see below).

Somewhat surprisingly, it turns out that whether or not a graph is “locally” common depends on the topology we consider on graph limits. This leads to (at least) two different versions of this notion: “locally common” and “weakly locally common.”

More recently Lovász [12] proved a “local” version of Sidorenko's conjecture, and characterized those graphs satisfying the weak local Sidorenko property [13]. If a graph is (locally, weakly locally) Sidorenko, then it is (locally, weakly locally) common, and so these (partial) results about the Sidorenko property have implications about common graphs by a similar reduction as (4). In particular, all bipartite graphs are locally common (Theorem 3.3 in [12]).

The goal of this paper is to show that every graph containing $K_{4}$ is locally common in the weakest sense, but not in a stronger sense. We give a rather general sufficient condition for a graph to be weakly locally common, and show that not all connected graphs are weakly locally common.

Very recently, a subsequent paper by Hancock et al. [8] made some further progress on characterizing weakly locally common graphs.

## 2 PRELIMINARIES

### 2.1 Graph limits

We need some definitions from the theory of graph limits; see [13] for more detail. A *kernel* is a symmetric bounded measurable function $W:{\left[0,1\right]}^{2}\to R$. (Instead of [0,1] we could use any other standard probability space here, and we shall do so if it is more convenient.) A *g*raphon is a kernel with values in [0,1]. We denote the set of kernels by W, the set of graphons by $W_{0}$, and the set of kernels with values in [−1,1] by $W_{1}$.

The significance of graphons is that they provide limit objects for convergent graph sequences. We call a sequence $\left(G_{1},G_{2},\text{…}\right)$ of (finite) simple graphs *convergent* if the numerical sequence $t\left(F,G_{n}\right)$ is convergent for every simple graph F [1]. It was proved in [14] that for every convergent graph sequence there is graphon W such that

$$t\left(F,G_{n}\right)\to t\left(F,W\right)\;\left(n\to \infty \right),$$

where for every graphon (or kernel) W we define

(5) $$t\left(F,W\right)=\int_{{\left[0,1\right]}^{V\left(F\right)}}\prod_{ij\in E\left(F\right)}W\left(x_{i},x_{j}\right)\prod_{i\in V\left(F\right)}dx_{i}.$$

Conversely, every graphon represents the limit of a convergent graph sequence.

These results make it possible to formulate our problems in a remainder‐term‐free form. A simple graph F is common if and only if

(6) $$t\left(F,W\right)+t\left(F,1-W\right)\geq 2^{1-|E\left(F\right)|}=2t\left(F,\frac{1}{2}\right)$$

for every graphon W (where 1/2 means the identically 1/2 graphon). We can multiply by $2^{|E\left(F\right)|}$, and write W=(1+U)∕2 (where $U\in W_{1}$) to get the inequality

(7) t(F,1+U)+t(F,1−U)≥2.

We call a simple graph F *locally common for perturbation* ε>0 if t(F,1+U)+t(F,1−U)≥2 for every $U\in W_{1}$ with $∥U{∥}_{\infty }\leq \varepsilon$. We say that F is *locally common* if there is an ε>0 such that F is locally common for perturbation ε.

A related notion is that the graph F is *weakly locally common* 1: this means that for every $U\in W_{1}$ there is an $\varepsilon_{U}>0$ such that t(F,1+εU)+t(F,1−εU)≥2 for all $0\leq \varepsilon \leq \varepsilon_{U}$.

It is clear that every common graph is locally common, and every locally common graph is weakly locally common. In the other direction, there are weakly locally common graphs which are not locally common, such as $K_{4}$, but we do not know any locally common graph which is not common.

Bipartite graphs are locally common (because locally Sidorenko [12]), but not known to be common. As cited above, Thomason [20] proved that the graph $K_{4}$ is not common, while Franek and Rödl [6] proved (in a different language) that $K_{4}$ is weakly locally common. It will follow from our results that $K_{4}$ is not locally common. Jagger et al. [10] proved that no graph containing $K_{4}$ as a subgraph is common. We are going to prove that a graph containing $K_{4}$ is always weakly locally common, but never locally common.

Similarly to common graphs, we can define “local” and “weakly local” versions of other extremal problems. We say that a simple graph F has the *local Sidorenko property for perturbation* ε if t(F,1+U)≥1 for every $U\in W_{1}$ with ∫U=0 and $∥U{∥}_{\infty }\leq \varepsilon$. It was proved in [12] that every bipartite graph F is locally Sidorenko for perturbation ε=1∕(4∣E(F)∣).

We call a simple graph F *weakly locally Sidorenko* if for every $U\in W_{1}$ with ∫U=0 there is an $\varepsilon_{U}>0$ such that t(F,1+εU)≥1 for every $0\leq \varepsilon \leq \varepsilon_{U}$. The weak local Sidorenko property is even easier to treat, as noted in [13], Section 16.5.3: *A simple graph has the weak local Sidorenko property if and only if it is a forest or its girth is even*.

These results immediately imply some facts about locally common graphs: every bipartite graph F is locally common for perturbation 1∕(4∣E(F)∣), and every graph with even girth is weakly locally common. We are going to prove a more general sufficient condition for being weakly locally common.

### 2.2 Subgraph densities

We call a graph *mirror‐symmetric* if it is obtained by the following construction: we take a graph G, select an independent set S⊆V(G), and glue together two copies of G along S.

The following simple facts have been noted in [3]. We note that the converse of the first assertion is conjectured in [3], which would provide a characterization to mirror‐symmetric graphs.

Lemma 2.1 If F is mirror‐symmetric, then t(F,U)≥0 for every kernel U. Furthermore, if F is an even cycle and U is not almost everywhere 0, then t(F,U)>0.

No matter how we fix the variables in the definition of t(F,U) corresponding to nodes in S, integrating the rest gives a square, which is nonnegative. The second statement follows from the fact that even cycles define a Schatten norm, see Section 14.1 in [13].   □

We say that a kernel U is *balanced* if $\int_{0}^{1}U\left(x,y\right)dy=0$ for almost all x∈[0,1]. Analogously, an edge‐weighted graph is *balanced*, if for every node v, the sum of weights of edges incident with v is 0.

Lemma 2.2 A kernel U is balanced if and only if $t\left(P_{3},U\right)=0$. If U is a balanced kernel, and a graph F has a node of degree 1, then t(F,U)=0.

Lemma 2.1 implies that $t\left(P_{3},U\right)\geq 0$ for every kernel U. The case of equality easily follows from the proof of the inequality.

If ${\text{deg}}_{F}\left(u\right)=1,uv\in E\left(F\right)$, then

$$\begin{array}{ccc} t\left(F,U\right) & \overset{\left(5\right)}{=} & \int_{{\left[0,1\right]}^{V\left(F\right)}}\prod_{ij\in E\left(F\right)}U\left(x_{i},x_{j}\right)\prod_{i\in V\left(F\right)}dx_{i}\\ & = & \int_{{\left[0,1\right]}^{V\left(F\right)⧹\left\{u\right\}}}\prod_{ij\in E\left(F\right)⧹\left\{uv\right\}}U\left(x_{i},x_{j}\right)\cdot \int_{0}^{1}U\left(x_{v},x_{u}\right)dx_{u}\prod_{i\in V\left(F\right)⧹\left\{u\right\}}dx_{i}=0 \end{array}$$

because $\int_{0}^{1}U\left(x_{v},x_{u}\right)dx_{u}=0$ for almost all $x_{v}$.   □

Let sub(H,F) denote the number of subgraphs of F without isolated nodes isomorphic to H. The densities in the “perturbed” graphons can be expanded:

(8) $$t\left(F,1+U\right)=\sum_{F\prime \subseteq F}t\left(F\prime ,U\right)=\sum_{H}\text{sub}\left(H,F\right)t\left(H,U\right).$$

Hence

(9) $$t\left(F,1+U\right)+t\left(F,1-U\right)=2\sum_{H:\;|E\left(H\right)|\text{even}\;}\text{sub}\left(H,F\right)t\left(H,U\right)=2+2p\left(F,U\right),$$

where

(10) $$p\left(F,U\right)=\sum_{H:\;\;0<|E\left(H\right)|\text{even}\;}\text{sub}\left(H,F\right)t\left(H,U\right).$$

Using this notation, we get the following rephrasing of the definitions of different versions of the common property.

Proposition 2.3

(a) A graph F is common if and only if p(F,U)≥0 for all $U\in W_{1}$.
(b) A graph F is locally common if and only if there is a number ε>0 such that p(F,εU)≥0 for all $U\in W_{1}$.
(c) A graph F is weakly locally common if and only if for every $U\in W_{1}$ there is a number $\gamma_{U}>0$ such that p(F,εU)≥0 for all $0<\varepsilon \leq \gamma_{U}$.

Defining

(11) $$c_{r}\left(F,U\right)=\sum_{H:|E\left(H\right)|=r}\text{sub}\left(H,F\right)t\left(H,U\right)\;\left(r=0,1,\text{…}\right),$$

we can express p(F,εU) as a polynomial in ε:

(12) $$p\left(F,\varepsilon U\right)=\sum_{r=1}^{\left\lfloor |E\left(F\right)|∕2\right\rfloor }\varepsilon^{2r}c_{2r}\left(F,U\right).$$

Using this expansion, assertion (c) in Proposition 2.3 can be rephrased as follows: *A graph* F *is weakly locally common if and only if for every* $U\in W_{1}$, *either* $c_{2}\left(F,U\right)=c_{4}\left(F,U\right)=\text{⋯}\;=0$, *or the first nonzero number in the sequence* $c_{2}\left(F,U\right),c_{4}\left(F,U\right),\text{…}$ *is positive*.

These observations give a short proof that the graph obtained from $K_{4}$ by deleting an edge is common. This was a result of Franek and Rödl [6], and a shorter proof with this language was presented in [13], Section 16.5.4.

## 3 LOCALLY COMMON GRAPHS

Our goal is to prove the following strengthening of the result of Jagger et al. [10], asserting that graphs containing $K_{4}$ are never common.

Theorem 3.1 No graph containing $K_{4}$ is locally common.

We start with some general consequences of the expansion formulas in the previous section. Let us introduce two operations on kernels: for a kernel U and 0<δ≤1, define a kernel $U_{\delta }\in W_{1}$ by

$$U_{\delta }\left(x,y\right)=\left\{\begin{array}{ll} U\left(x∕\delta ,y∕\delta \right), & \;\text{if}\;\;x,y\leq \delta ,\\ 0, & \;\text{otherwise}\;. \end{array}\right.$$

For a kernel U and positive integer m, we define the “tensor power” kernel $U^{⊗m}:{\left[0,1\right]}^{m}\times {\left[0,1\right]}^{m}\to \left[-1,1\right]$ by

$$U^{⊗m}\left(\left(x_{1},\text{…},x_{m}\right),\left(y_{1},\text{…},y_{m}\right)\right)=U\left(x_{1},y_{1}\right)\text{⋯}U\left(x_{m},y_{m}\right).$$

(Note that the measure spaces of [0,1] and ${\left[0,1\right]}^{m}$ are isomorphic, so we can rightfully call $U^{⊗m}$ a kernel.) It is straightforward that if $U\in W_{1}$ is balanced, then so are $U_{\delta }$ and $U^{⊗m}$. Furthermore, $t\left(F,U_{\delta }\right)=\delta^{|V\left(F\right)|}\;t\left(F,U\right)$ and $t\left(F,U^{⊗m}\right)=t{\left(F,U\right)}^{m}$. (We will use an odd m in this construction, so that the sign of t(F,U) is preserved.)

Substituting these expressions, we get the expansion

(13) p(F,ε(U⊗m)δ)=∑H: 0<∣E(H)∣evensub(H,F)ε∣E(H)∣δ∣V(H)∣t(H,U)m=∑q=2∣V(F)∣δq∑H:∣E(H)∣even∣V(H)∣=qsub(H,F)ε∣E(H)∣t(H,U)m.

Suppose that F is locally common for perturbation ε. Then p(F,εU)≥0 for every kernel $U\in W_{1}$, including every kernel of the form ${\left(U^{⊗m}\right)}_{\delta }$. The parameter ε is fixed, but we can play with the parameters δ and m.

Letting δ→0, we get that the first nonzero term in the outer sum must be positive. There is only one term with q≤3, namely $H=P_{3}$, and by Lemma 2.2, $t\left(P_{3},U\right)>0$ unless U is balanced. So let us assume that U is balanced. Then Lemma 2.2 implies that only those terms are nonzero where all degrees in H are at least 2. There are only two such graphs with q=4, namely $H=C_{4}$ and $H=K_{4}$. Thus (simplifying by $\delta^{4}\varepsilon^{2}$) we get a necessary condition for being locally common for perturbation ε:

(14) $$\forall \;\text{balanced}\;\;U\in W_{1}:\;\text{sub}\left(C_{4},F\right)t{\left(C_{4},U\right)}^{m}+\varepsilon^{2}\text{sub}\left(K_{4},F\right)t{\left(K_{4},U\right)}^{m}\geq 0.$$

We know from [15] that $t\left(C_{4},U\right)>0$ for every U≢0, so the condition is trivially satisfied if F contains no $K_{4}$. Our goal is to prove the converse.

Letting m→∞, this implies that

(15) $$t\left(C_{4},U\right)\geq \left\{\begin{array}{ll} -t\left(K_{4},U\right), & \;\text{if}\;\;\text{sub}\left(K_{4},F\right)>0,\\ 0, & \;\text{otherwise}\;. \end{array}\right.$$

This strange conclusion, which is independent of ε and almost independent of F, says the following: either $t\left(C_{4},U\right)+t\left(K_{4},U\right)\geq 0$ for every balanced $U\in W_{1}$, or no locally common graph contains $K_{4}$. We show that the second alternative occurs, by constructing a kernel U violating the first inequality. The construction is carried out in several steps.

**Figure 1:**
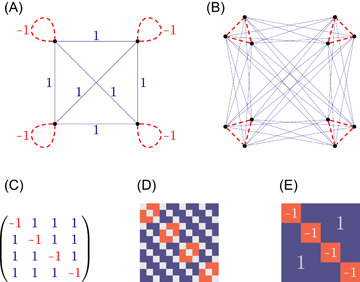
(A) *G*_1_, (B) *G*_2_ = *K*_3_ × *G*_1_, (C) *G*_1_, (D) *G*_2_, and (E) $\underset{n\to \infty }{\lim\;}K_{n}\times G_{1}$. [Color figure can be viewed at wileyonlinelibrary.com]

Claim 1 There exists a looped‐simple graph $G_{1}$ with edgeweights ±1 such that

$$t\left(C_{4},G_{1}\right)+t\left(K_{4},G_{1}\right)=-1∕4.$$

Let $G_{1}$ obtained from $K_{4}$ by adding a loop with weight −1 at every node. By direct calculation, $t\left(C_{4},G_{1}\right)=1∕4$ and $t\left(K_{4},G_{1}\right)=-1∕2$, thus $t\left(C_{4},G_{1}\right)+t\left(K_{4},G_{1}\right)=-1∕4$.

Claim 2 There exists an arbitrarily large simple graph $G_{2}$ (without loops) with edgeweights ±1 such that $t\left(C_{4},G_{2}\right)+t\left(K_{4},G_{2}\right)\leq -1∕5$.

Indeed, consider any looped‐simple graph $G_{1}$ with the properties of Claim 1, and take its categorical product $G_{2}=K_{n}\times G_{1}$, where $K_{n}$ is a large complete graph (without loops). Then $G_{2}$ has no loops, and

$$\begin{array}{cc} t\left(C_{4},G_{2}\right)+t\left(K_{4},G_{2}\right) & =t\left(C_{4},K_{n}\right)t\left(C_{4},G_{1}\right)+t\left(K_{4},K_{n}\right)t\left(K_{4},G_{1}\right)\\ & \to \;t\left(C_{4},G_{1}\right)+t\left(K_{4},G_{1}\right)=-\frac{1}{4}\;\left(n\to \infty \right). \end{array}$$

So $t\left(C_{4},G_{2}\right)+t\left(K_{4},G_{2}\right)\leq -1∕5$ if n is large enough.

In Figure 1A,B, solid blue lines indicate edges with weight 1, red dashed lines indicate edges with weight −1. Figure 1C,D show their adjacency matrices, blue, gray, and red represent 1, 0, −1, respectively. In Figure 1B,D, we used *n* = 3, namely, *G*_2_ = *K*_3_ × *G*_1_. In the language of graph limits, Figure 1E shows the graphon of *K_n_* × *G*_1_ in the limit n→∞.

Claim 3 There exists a simple graph $G_{3}$ with balanced edgeweights ±1 such that $t\left(C_{4},G_{3}\right)+t\left(K_{4},G_{3}\right)<0$.

Let $G_{2}$ be a graph in Claim 2, and let $V\left(G_{2}\right)=\left[r\right]$. Note that r can be arbitrarily large.

**Figure 2:**
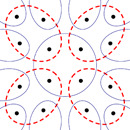
The local structure of the hypergraph H for r=4. The hyperedges $A_{i}$ and $B_{j}$ are shown by blue solid ellipses and red dashed ellipses, respectively [Color figure can be viewed at wileyonlinelibrary.com]

Consider an r‐regular bipartite graph with a girth at least 5. (A positive fraction of r‐regular bipartite graphs on n→∞ vertices has a large girth.) Its dual (see Figure 2) is an r‐uniform r‐partite hypergraph H (the r‐partitioning corresponds to a proper r‐edge coloring of the original graph).

This r‐uniform hypergraph has two families of edges $\left\{A_{1},\text{…},A_{N}\right\}$ and $\left\{B_{1},\text{…},B_{N}\right\}$ such that the sets $A_{i}$ as well as the sets $B_{i}$ form a partition of V(H), and H has girth at least 5. Let $V_{1},\text{…},V_{r}$ be the partition classes of H. We glue a copy of $G_{2}$ on every $A_{i}$ and every $B_{i}$ (node u of $G_{2}$ is glued onto the node of $A_{i}$ in $V_{u}$). In the sets $A_{i}$, we keep the original weighting of the edges; in the sets $B_{i}$, we multiply them by −1.

It is clear that the weighted graph $G_{3}$ constructed this way is balanced. Furthermore, every homomorphism $K_{4}\to G_{3}$ maps $K_{4}$ into one of the $A_{i}$ or into one of the $B_{i}$, and hence $\text{hom}\left(K_{4},G_{3}\right)=2N\;\text{hom}\left(K_{4},G_{2}\right)$. This is not quite true for $C_{4}$ in place of $K_{4}$, but the difference is small: it counts those homomorphisms $C_{4}\to G_{3}$ for which two opposite nodes of $C_{4}$ are mapped onto the same node v of $G_{3}$, and the other two nodes are mapped into different copies of $G_{2}$ containing v. Hence

$$\text{hom}\left(C_{4},G_{3}\right)-2N\;\text{hom}\left(C_{4},G_{2}\right)\leq 2r^{3}N,$$

and for r>5,

$$\begin{array}{ccc} t\left(C_{4},G_{3}\right)+t\left(K_{4},G_{3}\right) & = & \frac{1}{r^{4}N^{4}}\left(\text{hom}\left(C_{4},G_{3}\right)+\text{hom}\left(K_{4},G_{3}\right)\right)\\ & \leq & \frac{1}{r^{4}N^{3}}\left(2\;\text{hom}\left(C_{4},G_{2}\right)+2\;\text{hom}\left(K_{4},G_{2}\right)+2r^{3}\right)\\ & = & \frac{2}{N^{3}}\left(t\left(C_{4},G_{2}\right)+t\left(K_{4},G_{2}\right)+\frac{1}{r}\right)<0. \end{array}$$

This proves Claim 3. □

## 4 WEAKLY LOCALLY COMMON GRAPHS

We have seen that every forest and every graph with even girth is weakly locally common. We prove more in the next theorem. We define $g_{\text{even}}\left(F\right)$ as the length of the shortest even cycle of F, where $g_{\text{even}}\left(F\right)=\infty$ if F has no even cycle.

Theorem 4.1 If F is not weakly locally common, then F has two odd cycles with at most one node in common, of lengths $g_{1}$ and $g_{2}$, such that either

- $g_{1}<g_{2}$ and $g_{1}+g_{2}\leq g_{\text{even}}\left(F\right)$, or
- $g_{1}=g_{2}$ and $g_{1}+g_{2}<g_{\text{even}}\left(F\right)$.

In particular, if the length of the shortest even cycle in F is at most twice the length of the shortest odd cycle in F, or F has no odd cycle, then F is weakly locally common.

Suppose that F is not weakly locally common. By (12) it means that the sequence $c_{2}\left(F,U\right),c_{4}\left(F,U\right),\text{…}$ has a nonzero term, and its first nonzero term, say $c_{2p}\left(F,U\right)$, is negative. We know that $c_{2}\left(F,U\right)\geq 0$ by Lemma 2.1, so p>1. Hence $c_{2}\left(F,U\right)=0$, which implies that U is balanced. In this case, t(H,U)=0 for every graph H having a node of degree 1 by Lemma 2.2.

Let r be the smallest positive integer for which F has a subgraph H with 2r edges and t(H,U)<0 (the inequality $c_{2p}\left(F,U\right)<0$ implies that such a subgraph exists and r≤p). We know by the above that 2≤r. Lemma 2.2 implies that all degrees in H are at least 2. We have t(F′,U)=0 for every subgraph F′ of F with ∣E(F′)∣<2r and ∣E(F′)∣ even. In particular, by Lemma 2.1 there cannot be any even cycle in F with length less than 2r, thus $g_{\text{even}}\left(F\right)\geq 2r$. Moreover, t(H,U)<0 implies that H itself is not an even cycle, Lemma 2.1 implies that $g_{\text{even}}\left(F\right)\geq 2r$ and since H is not an even cycle, it cannot contain an even cycle.

It is a well‐known elementary exercise that every block (2‐connected component) in a graph with no isolated vertices and even cycles is either an odd cycle or an edge. Therefore, H cannot be 2‐connected. Let $C_{1}$ and $C_{2}$ be two leaves in the block‐cut tree of H; these cannot be edges, otherwise H would contain a leaf. Thus $C_{1}$ and $C_{2}$ must be two odd cycles in H of lengths $g_{1}$ and $g_{2}$ intersecting in at most one node. Therefore, $g_{1}+g_{2}\leq |E\left(H\right)|=2r\leq g_{\text{even}}\left(F\right)$.

To complete the proof, we have to exclude the case $g_{1}=g_{2}=r$. In this case $H=C_{1}\cup C_{2}$, and H is mirror‐symmetric, which implies by Lemma 2.1 that t(H,U)≥0.   □

Corollary 4.2 Every graph containing $C_{4}$ or $C_{6}$ is weakly locally common.

So far we have only shown positive results for a graph to be weakly locally common. This motivates the following proposition.

Proposition 4.3 There exist connected graphs that are not weakly locally common.

**Figure 3:**
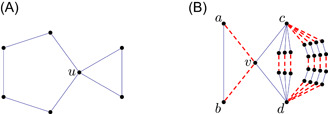
(A) The graph F and (B) the graph G with k=3. [Color figure can be viewed at wileyonlinelibrary.com]

Let F consist of a triangle and a pentagon, attached to each other at one node u (Figure 3A). We construct a balanced edge‐weighted graph G with edgeweights ±1 such that t(F,G)<0 (Figure 3B) We start with a 4‐star with center node v and endnodes a,b,c,d. Let k be a large positive integer. We connect a and b by an edge; we attach k openly disjoint paths $Q_{1},\text{…},Q_{k}$ of length 3 and k+1 further openly disjoint paths $R_{1},\text{…},R_{k+1}$ of length 5 connecting c and d. We weight the following edges with −1: the edges va and vb; the middle edge of every path $Q_{i}$; and every second edge of each path $R_{i}$, starting at the end. The remaining edges are weighted with 1. It is clear that the weighting is balanced.

We claim that

(16) t(F,G)<0.

The normalization is irrelevant, so it suffices to show that hom(F,G)<0. Let ϕ:V(F)→V(G) be a homomorphism. The triangle in F must be mapped onto the triangle in G. If the pentagon in F is mapped into the subgraph G[S] induced by S={v,a,b,c,d}, then the contribution of ϕ is positive, but the number of these maps is independent of k (52, in fact). If the image of the pentagon contains a node outside S, then it must contain one of the paths $Q_{i}$, and then u must be mapped onto v. The contribution from such a map is −1, and the number of such maps is 4k. Thus hom(F,G)=52−4k, which is negative if k>13. This proves (16).

The condition that G is balanced implies that $t\left(F\prime ,W_{G}\right)=0$ if F′ has a node with degree 1. The only subgraph of F with an even number of edges and with all degrees at least 2 is F itself, and hence $c_{2}\left(F,W_{G}\right)=c_{4}\left(F,W_{G}\right)=c_{6}\left(F,W_{G}\right)=0$ but $c_{8}\left(F,W_{G}\right)=t\left(F,W_{G}\right)<0$. Thus F is not weakly locally common. □

## 5 OPEN PROBLEMS

In the definition of locally common graphs, we can consider various norms on the space W instead of the $L_{\infty }$ norm. Can the results above be extended to other norms? An important candidate is the *cut norm*, defined by

$${||W||}_{□}=\underset{S,T\subseteq \left[0,1\right]}{\text{sup}}\left|\int_{S\times T}W\left(x,y\right)\;dx\;dy\right|,$$

playing an important role in the theory of graph limits. It was proved in [12] that every bipartite graph is locally Sidorenko with respect to the cut norm. Since the cut norm is continuous with respect to every “reasonable” norm on W (for an exact formulation of this fact see [13], Theorem 14.10), it follows that every bipartite graph is locally Sidorenko in every “reasonable” norm on W.

Similar to common graphs and Sidorenko graphs, we can define “local” and “weakly local” versions of other extremal properties (graph homomorphism inequalities), but little is known in this direction.

Are there any noncommon graphs that are locally common in the cut norm or the $L_{\infty }$ norm? Is there a graph that is locally common with respect to the $L_{\infty }$ norm, but not with respect to the cut norm? Can weakly locally common graphs be characterized similarly as weakly locally Sidorenko graphs?

## Data Availability

The data that support the findings of this study are openly available in no data at https://arxiv.org/abs/1912.02926.

## Footnotes

1 In [13], only this version was defined and called “locally common.”
